# Multi-QuadEmoNet: cat and dog emotion classification model from animal vocalization using multi-stage LSTM-GRU paradigm

**DOI:** 10.3389/fvets.2026.1799281

**Published:** 2026-06-22

**Authors:** Sundara Sobitha Raj Anubha Pearline, Geetha Subbiah, Ashley Sanu, Karunamayi Charumathi Venkatesan

**Affiliations:** School of Computer Science and Engineering, Vellore Institute of Technology Chennai Campus, Chennai, Tamil Nadu, India

**Keywords:** animal emotion recognition, deep learning, long short-term memory, Mel-Frequency Cepstral Coefficients, QuadEmoNet

## Abstract

**Introduction:**

Decoding the emotional nuances within animal vocalizations poses a significant challenge due to their intricate nature and the limitations of traditional analysis methods. Pets enrich our lives, but true companionship requires recognizing their emotional states. By deciphering signs of happiness, fear, or anxiety, humans interact to nurture a positive relationship with the pet animals.

**Method:**

This research aims to address this challenge by developing a multi-modal approach for animal emotion voice recognition system using the proposed Multi-Quadro-Emotion Network (Multi-QuadEmoNet) method. The proposed QuadEmoNet was a neural network specifically designed for handling audio data. Initially, Mel-Frequency Cepstral Coefficients (MFCCs) were used to extract features from the preprocessed audio data. The extracted features were used to train the QuadEmoNet deep learning model for animal emotion classification. Multi-QuadEmoNet contains three QuadEmoNets. The first QuadEmoNet was trained initially to predict cat/dog. Further, second and third QuadEmoNet’s were implemented to classify cat and dog emotions separately after training the model for 50 epochs.

**Results and discussion:**

The proposed system utilizes a self-collected dataset containing 6,000 cat audio samples and 2000 dog audio samples. The designed QuadEmoNet model was evaluated producing highest accuracy of 95% (DMAD), and an accuracy of 90% (CMAD) was achieved for 75 and 50 epochs. Based on the proposed Multi-QuadEmoNet model, an user interface for recognizing the animal emotions was developed for predicting the animal emotions.

## 1 Introduction

Emotions are a state of feeling of pleasure or displeasure, thoughts in relation to the events happening in our day-to-day lives. Similar to human emotions, animals also possess a wide range of emotions related to their living area. Some basic feelings of human beings are fear, anguish, happy, sad. Similarly, animals also have emotions. Animal emotions can be analyzed using audio spectrums. Animal sound recordings are analog signals whose frequency components are identified through spectrograms.

Emotions in animals are evolutionarily inherited mechanisms that regulate behavior, decision-making, and survival across species. Neurobiologically, these processes are driven by interconnected structures of the limbic system, which combines external stimuli with physiological responses to generate adaptive behaviors. Emotional states are communicated through multimodal signals, including vocalizations, facial expressions, and body postures, enabling both intra- and interspecific interactions (1–4). Such expressions provide reliable indicators of internal affective states and social intentions. Importantly, emotions are attentively linked to animal welfare, as both positive and negative states reflect the quality of environmental and physiological conditions. Behavioral patterns, including feeding, chewing, and vocal activity, serve as practical proxies for assessing health and well-being. Hence, incorporating behavioral and neurobiological insights supports the use of computational approaches for objective evaluation of animal emotions.

Animal emotions have become a popular topic in biological studies because they are crucial for human understanding of animal behavior, but they are also important for animal welfare and, consequently, for policymakers (4). Nowadays, it is widely recognized that mammals, other vertebrates, and even certain invertebrates have emotions that are quite comparable to some of our own. These emotions are thought to have adaptive importance as they enable these creatures to react to different situations in a timely and appropriate manner, thereby helping their survival.

Pet owners interpret animals’ emotions. The pet companionship owners regard their pets as members of their own family. The feelings of human companions and pet animals express the association between them (5). The owners state that animals have basic feelings such as joy, fear, surprise, and fury, while pet animals exhibit complicated emotions such as empathy, shame, or disappointment.

Emotions can activate behavioral and physiological mechanisms in response to stimuli, enabling animals to escape damage or aim for resources, consequently enhancing their chances of survival (5, 6). The use of electrical stimulation of the brains of both humans and nonhuman animals has shown that all mammals have identical brain architecture and unconditioned emotional reactions (5). As a result, it has been shown that emotions can be understood and mirrored both within and between species (6). Emotion transmission occurs when animals perceive another animal’s emotional state, such as fear, and modify their own affective state accordingly (7).

Emotions in animals are evolutionarily conserved mechanisms that support survival by enabling adaptive responses to threats, social interactions, and environmental changes. These states are communicated through multimodal signals, including vocalizations, body posture, and facial expressions, which convey information about arousal and valence. Among these, vocalizations are particularly effective due to their rapid transmission and ability to operate over distance. Prior research shows that acoustic features such as frequency, intensity, and temporal structure are closely linked to emotional states, supporting vocal analysis as a reliable and non-invasive approach for assessing animal emotions.

The fields of ethology and artificial intelligence are amalgamated because of the growing importance of understanding the emotional states of pet animal friends via human-animal interactions. This research presents a novel initiative to unravel the complex emotions found in domesticated animals’ vocalizations, with a particular focus on dogs and cats. This research aims to create a sophisticated system that can identify and categorize various emotional states-mainly sadness and anger-that these animals communicate through their vocalization.

Interpreting the emotions of animal vocalizations (7, 8) was a complex task. Numerous factors influence animal noises; even a cat’s meow was a complex puzzle for laymen to understand their emotions. Also, a cat’s happy greeting emotion might elicit a high-pitched purr, while fear might trigger a harsh yowl. Age, breed, and personality all contribute to these variations. Interspecies and intraspecies similarities present challenges when dealing with animal vocal-based emotion recognition.

This research tackles these complexities by combining cutting-edge deep learning with sophisticated audio processing techniques (9). Deep learning was quite effective at identifying intricate patterns in data. Deep learning models may recognize small differences that correspond to particular emotions by being trained on a large collection of tagged sounds made by animals. Long Short-Term Memory (LSTM) (9) was a type of Recurrent Neural Network (RNN) (10) designed to tackle sequential data like audio signals.

Long-term storage of information was made possible by the specialized units of LSTM architecture known as memory cells. LSTMs possess the ability to selectively recall or forget data according to the context of the input sequence. This was crucial because animal vocalizations often carry emotional cues that unfold over time. For instance, a dog’s whine might gradually escalate into a full-blown howl if left unattended, indicating increasing distress. An LSTM can capture this progression in the audio by analyzing the sequence and recognizing the emotional shift. Owing to the difficulties that are presented with the diversity and complexity of animal vocalizations based on emotion recognition, this research approach combines cutting-edge deep learning technologies and pre-processed audio signal processing techniques.

Raw audio data is similar to an external language to the model. Hence, the authors have pre-processed and extracted features using Mel-Frequency Cepstral Coefficients (MFCC) (11). The rationale for this decision stems from MFCC’s capacity to extract crucial spectral properties and express them as compact features, which makes it easier to identify emotional fingerprints. The main aim of this paper was to identify the distinct patterns that underpin auditory emotional responses through an exploration of the tonal nuances of animal vocalizations. Given their essential roles in human families and the close emotional relationships they share with their owners, the use of dogs and cats as study subjects was especially noteworthy. Examining these companion animals’ emotional subtleties advances the science of affective computing and has implications for improving human comprehension of interspecies communication and developing compassionate relationships.

The major contributions of this paper are:

- Creation of self-collected Animal Audio Datasets: Dog Mood Audio Dataset (DMAD) and self-collected Catus Mood Audio Dataset (CMAD). DMAD and CMAD contains self-created dog and cat audio emotions.
- Multi-QuadEmoNet (Multi-Quadro Emotion Network) was developed for categorizing pet animal emotions. Two stage QuadEmoNet was utilized for animal audio emotion classification. The first level of QuadEmoNet classifies if the audio was dog or cat. At the second level, two separate QuadEmoNets assist in classifying dog or cat emotions. Each and every layer of QuadEmoNet extract minute details of dog/cat animal audio sensory inputs.
- Assessing the performance of the two animal audio datasets using various Machine learning, deep learning, and Multi-QuadEmoNet.
- Implementation of the cat and dog audio-based real-time emotion identification using an interactive website.

The paper is organized as introduction, followed by literature review. In the third section of this paper, the proposed QuadEmoNet method was discussed. Further, in the fourth section, a detailed analysis of the proposed method, machine learning and deep learning methods are discussed. Finally, the conclusion section summarizes the paper.

## 2 Literature review

The field of emotion recognition has experienced a surge of innovation in recent years. Researchers have explored the intricate facets of emotional expression across a wide range of animal species. Three subsections, namely, 2.1–2.3 discuss on the innovative methodologies employed to decode the emotional nuances of human, cat/dog, and other animal vocalizations. Also, the challenges or problems faced in the existing literatures are summarized at the end of this section.

### 2.1 Human emotion recognition

Lazli et al. (12) focused on speaker-independent isolated speech recognition for the Arabic language using a hybrid HMM-MLP-FCM system. The study compared the performance of standard Hidden Markov models (HMM) with hybrid approaches that incorporate Multilayer Perceptron (MLP) to estimate HMM emission probabilities. The primary objective of the authors was to evaluate and compare the performance of different approaches for both task-dependent and task-independent training. The hybrid HMM/MLP system utilized the Fuzzy C-Means (FCM) algorithm to segment acoustic vectors, enhancing the accuracy and robustness of the recognition system. By leveraging both HMM and MLP techniques along with FCM-based segmentation, the study aimed to enhance the efficiency and accuracy of speaker-independent Arabic speech recognition.

Jain et al. (13) suggested a robust system employing MFCC and Linear Predictive Cepstral Coefficients (LPCC) for feature extraction and Support Vector Machine (SVM) for classification. The two SVM classification strategies, One-against-All (OAA) and Gender-Dependent Classification, were explored, providing valuable insights into their effectiveness for human emotion recognition. Despite focusing on four basic emotions, the paper contributes to understanding the suitability of algorithms for this complex task.

Liu et al. (14) introduced an innovative method for speech emotion recognition, incorporating feature selection based on correlation analysis and Fisher’s criterion. The Extreme Learning Machine (ELM) decision tree achieves high accuracy (89.6%) across six emotions, showcasing efficiency in emotion recognition. The study stands out addressing the critical role of feature selection in optimizing model performance.

Hema et al. (15) delved into Emotional Speech Recognition (ESR) using CNN and Deep Learning models. The study emphasizes the spectral and prosodic aspects of audio signals, employing MFCC. Their proposed SER system attained an accuracy rate of 78%, underscoring the significance of integrating advanced machine-learning models for improved accuracy and efficiency in emotion recognition systems.

Van et al. (16) focused on human emotional expressions, leveraging deep neural networks, including CNN, Convolutional Recurrent Neural Network (CRNN) and Gated Recurrent Unit (GRU). The study attained an exceptional average recognition accuracy of 97.47%, positioning it as a significant advancement in the field of speech emotion recognition. The exploration of emotional expressions in voice highlights the universal nature of emotional communication.

Tellai et al. (17) introduced CNN-Transformer fusion approach for analyzing human speech based emotions. Initially, the authors fed the speech audios into MFCC and applied the fusion approach. This approach used for classification was capable of anal yzing spatial (CNN) and temporal (transformer) information of the audio signals. For realizing human emotions using Artificial Intelligence, Tellai and Mao (18) introduced Contextualized Convolutional Transformer-GRU Network (CCTG-Net). All human emotion audios were extracted with MFCC features. Further, the features were passed onto CCTG-Net. The authors evaluated the method using IEMOCAP dataset and their method produced an accuracy of 88%. Tellai et al. (19) proposed a speech emotion recognition system for analyzing specifically IEMOCAP dataset. The authors extracted features using MFCCs and classified the features using hierarchical attention model. The authors obtained an accuracy of 75% for the hierarchical attention method.

Verde et al. (20) addressed the voice disorders using machine learning algorithms. The authors explored mobile health systems for voice pathology detection, achieving high accuracy with SVM and DT algorithms. The proposed m-Health system holds promise for real-time voice disorder identification, emphasizing the importance of technological advancements in addressing voice disorders. Ayvaz et al. (21) explored the realm of Automatic Speaker Recognition (ASR) using MFCC and machine learning. Also, authors distinguished Turkish speakers using MFCCs and various machine learning algorithms. The MLP with a Tanh activation function achieves the highest accuracy at 90.2%, showcasing the effectiveness of MFCCs in ASR systems.

Ismail et al. (22) proposed a technique for speaker classification addressing the need for voice biometrics. The authors created their own voice datasets. Further, applied machine learning algorithm to the dataset. Artificial Neural Network (ANN) outperformed other machine learning classifiers. Singh et al. (23) identified the speaker based on audios. Features were extracted using PCA. For classification, SVM and k-Nearest Neighbor classifiers are employed.

Al-Anzi et al. (24) investigated the prominence of the MFCC method in Automatic Speech Recognition (ASR) systems. The authors extracted feature vectors using MFCC. The authors underscored MFCC’s dominance in Arabic ASR research based on prior studies, emphasizing its role as the standard choice for front-end features in contemporary ASR systems. The author concluded advocating for future research to compare MFCC with alternative methods such as Linear Predictive Coding Coefficients (LPCC) and Perceptual Linear Prediction (PLP).

Chu et al. (25) addressed the challenge of recognizing environmental sounds to understand the context surrounding an audio sensor. While MFCCs are commonly used for audio recognition, they may not adequately capture the temporal aspects of environmental sounds, which often have strong temporal domain signatures. To address this, the authors proposed utilizing the matching pursuit (MP) algorithm to extract effective time-frequency features from environmental sounds. By choosing features from an atom dictionary, the MP-based approach generates a collection of features that are both adaptable and comprehensible. By combining these MP features with MFCC features, the recognition accuracy for environmental sounds was significantly improved.

Totakura et al. (26) employed mathematical carriers such as Fourier transformations for Mel-Frequency Cepstral coefficients (MFCC). The study integrated neural networks with a layered architecture utilizing Rectified Linear Units (ReLU), achieving an impressive accuracy of 88%. This study showed that machine learning was effective in this field. Also, it anticipated practical implications for understanding and decoding animal emotions.

### 2.2 Cat and dog emotion recognition

In the domain of intelligent pet monitoring, Tsai et al. (6) introduced a novel approach to pet sentiment analysis using deep learning technologies within the Internet of Things (IoT) framework. The study achieved a remarkable 70% increase in accuracy compared to traditional methodologies, emphasizing the potential impact of deep learning in capturing nuanced features for pet sentiment analysis. The integration of posture analysis and sound signals showcases the potential for advanced technology in intelligent pet monitoring systems.

Ntalampiras et al. (27) proposed pre-trained YAMNet for categorizing cat vocalizations. The authors have processed audio signals and fed them into Short-time Fourier Transform (STFT). Further, Mel-spectrograms were obtained.

Also, it was passed into Depthwise convolutional architecture called YAMNet. This architecture follows VGGNet (Very Large Convolution Network), and it has lesser weights compared to VGGNet. The authors have trained the model on Adam optimizer. The authors have also created an app called MeowTalk for recognizing cat emotions.

Sinnott et al. (28) introduced a self-created Convolutional Neural for identifying barking dog behavior. The authors have used the ESC-50 dataset. The dataset was pre-processed and features were extracted using MFCC. The extracted features as images are passed as a multi-input CNN model. This multi-input CNN has two networks. The first network contains two convolution layers along with max pooling. The authors have used a second network consisting of two layers with Mel spectrogram features as input. Both the networks were concatenated and were followed by two fully connected layers. For the binary classification of dog barking, the authors obtained an accuracy of 80% approximately.

Quaranta et al. (29) compared human and cat emotions to analyze the correlation between them. Cat emotions are captured through audio recordings and facial images. Similarly, human emotions, such as happiness and anger, were recorded using auditory signals. The authors performed this analysis to explore the relationship between the acoustic and visual emotional signals of humans and animals.

Sun et al. (30) proposed Purrai for identification of cat’s language expressed through their sounds. Purrai was a neural network forming the base of Very Large Convolutional Neural Network (VGGNet) and helps in classifying nine various cat sounds. At the secondary level, the cat’s emotion (as sentence) was classified using 1D-Convolutional Neural Network (1D-CNN). The author’s network, “Purrai” produced an accuracy of 81.1%.

Tang et al. (31) designed Joint Learning-Time Frequency domain information and Multi-Scale Features Network (JL-TFMSFNet). Using their method’s Multi-Scale Feature Extraction module, mel-filter features were obtained from cat audios. CotNet was included in the network to fuse the features. Time-Frequency Attention Mechanism module was used to obtain semantic information from Mel features. Diverse Branch Block was utilized to recognize the cat sounds. JL-TFMSFNet was tested on Urbansound8K and their self-collected Cat Emotion Sound datasets.

Perez et al. (32) analyzed and identified the dog breed types based on their barks using machine learning and deep learning approaches. Initially, the recorded audios are passed to obtain the MFCC features. The significant features were identified using two evaluator and search methods namely, Relief Attribute Evaluation with Ranker and Subset evaluation with best first. Further, decision tree, SVM, Random Forest, Bagging and ConvNet are applied to identify their performance. The method was tested on their self-collected dataset consisting of 6,103 dog bark records of various dog breeds. F1-score value of 90.5% was achieved using SVM classifier.

### 2.3 Other animal emotion recognition

Mehdizadeh et al. (33) introduced an innovative approach to understanding nutritional behavior in dairy cattle. The approach received classification predictions ranging from 89.62 to 95.9% using various classifiers. Inherently, the cows monitored exhibit emotions such as hunger, calm and stress. Shi et al. (34) pioneered advancements in livestock health monitoring. The authors utilized preprocessing techniques, including double threshold endpoint detection and MFCC extraction. This method helped in achieving accurate recognition of pig cough sounds. Pig expresses emotions such as fear, contentment and isolation.

Deep learning-based livestock voice classification for real-time cattle monitoring was suggested by Jung et al. (35). The study employed CNN models and Short-Time Fourier Transform (STFT)-based noise filtering. Their method achieved an accuracy of 94.18% in classifying cattle sounds. The web-based monitoring system offered practical insights for farm owners, promoting efficient and humane livestock management practices. Using machine learning technology to store and categorize bird cries, Mehyadin et al. (36) developed a revolutionary bird classifier system. Their study utilized MFCC and various algorithms. Using their method, an accuracy of 94.18% was attained in bird sound classification.

Li et al. (37) addressed the poultry industry’s critical need for early gender detection in chicks. The authors employed deep learning techniques and achieved high accuracy (95%) in chick gender detection. Experimental evaluations conducted on the Latin Music Database (LMD) and the WASMIR 2004 dataset demonstrate the superiority of texture features over conventional audio content-based features. On the LMD dataset, their approach achieved an impressive recognition rate of approximately 82.33%, outperforming previous state-of-the-art results. Similarly, on the WASMIR 2004 dataset, the recognition rate reaches 80.65%, placing it competitively among existing literature.

This extensive review exaggerates the diversity and innovation within the field of emotion recognition and vocal analysis. It was noted in the existing literature that no standard datasets are available specifically for cat and dog audio emotions. It was observed that in most of the existing literatures, irrespective of the living being audios, authors have employed MFCC for pre-processing and feature extraction. The existing literature addresses human speech and animal emotion identification; however, methods for recognizing pet animal behaviors are still constrained. Applications of deep learning, especially those integrating LSTM and GRU, are sparse in this field. To address this gap, we propose QuadEmoNet, which integrates the advantages of LSTM and GRU for simulating acoustic dynamics. Based on QuadEmoNet, our Multi-QuadEmoNet framework was built and utilizes three QuadEmoNets for efficient classification of cat and dog emotions, strengthened by our meticulously curated pet mood audio datasets [Catus Mood Audio Dataset (CMAD) and Dog Mood Audio Dataset (DMAD)]. Section 3 and 4 elaborate about the proposed methodology ‘Multi-QuadEmoNet’.

## 3 Proposed methodology

**Figure 1:**
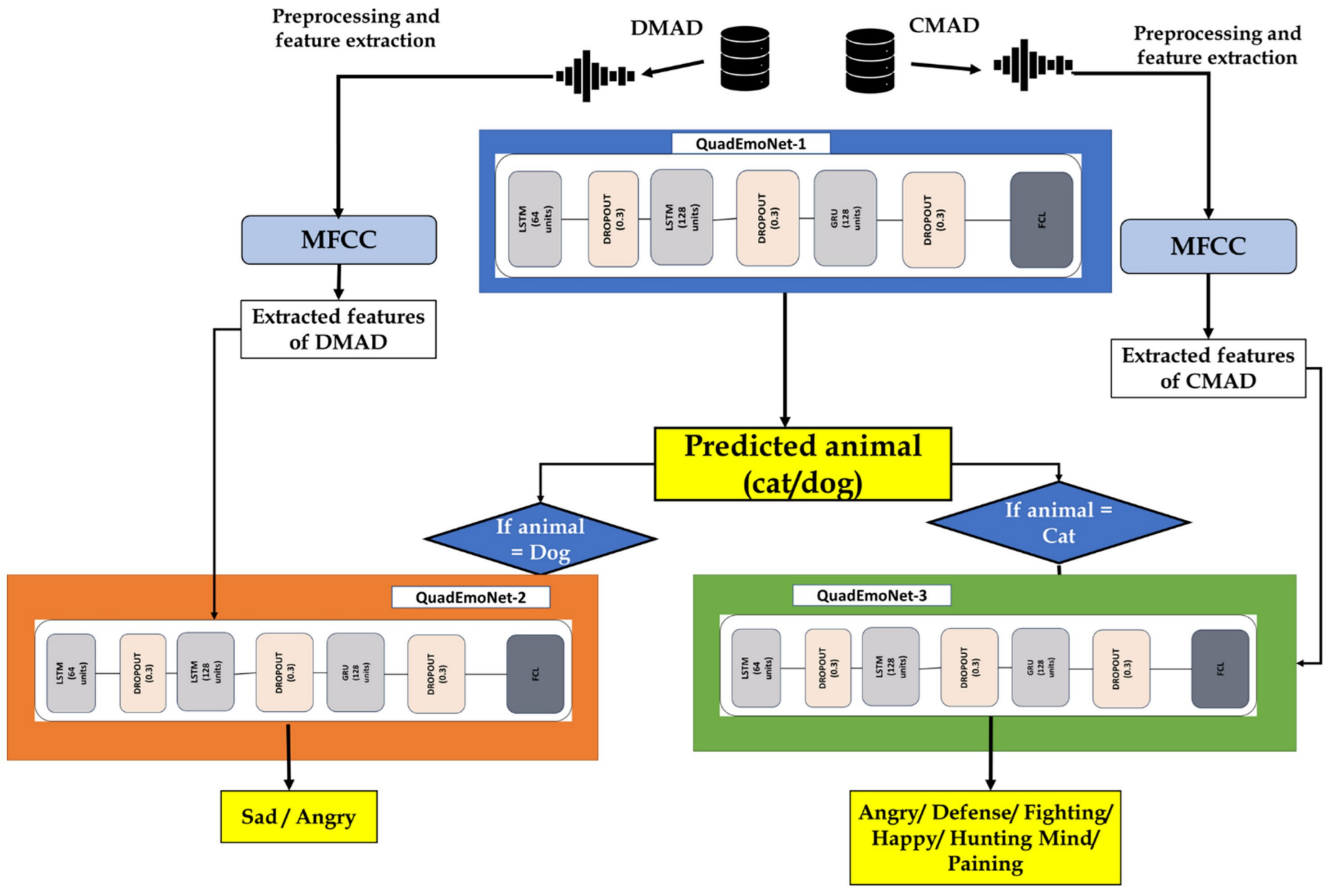
Proposed workflow for pet animal emotion recognition.

The proposed workflow methodology for pet animal emotion recognition was illustrated in Figure 1, portraying the animal voice-based emotion recognition model. The framework was operated based on a four-step process. First, we meticulously curate two datasets, the Dog Mood Audio dataset for dog audios and the Catus Mood Audio dataset for cat audios encompassing a wide variety of animal vocalizations. This dataset serves as the foundation for our analysis. Subsequently, each audio sample undergoes Mel-frequency Cepstral Coefficient (MFCC) extraction. MFCCs essentially convert the raw audio into a visual representation, highlighting the animal sound’s unique characteristics. 40 MFCC features were extracted from each audio sample.

The proposed system uses 3 QuadEmoNet models. By feeding the MFCCs into the initial QuadEmoNet model, i.e., the QuadEmoNet model for animal classification, this model was trained to recognize if audio was from cat or dog. After predicting the animal, most suited emotion of cat or dog was classified based on the particular animal audio. For training the animal emotion models, at secondary level one QuadEmoNet model was trained for Dog emotion classification with the MFCCs obtained from the Dog Mood Audio dataset. Similarly, another QuadEmoNet model was trained for Cat emotion classification with the MFCCs obtained from the Catus Mood Audio dataset. Finally, the models’ effectiveness was rigorously evaluated by testing it with entirely new, unseen audio recordings. This crucial stage enables us to assess the model’s capacity to interpret animal sounds, such as a happy chirp, a distressed screech, or a fun growl, into their emotions and create human-pet bond.

### 3.1 Dataset collection

Two datasets were utilized for training the models for this research. Both datasets consists of 100 audio samples for each emotion of both cats and dogs. Audios were obtained from diverse sources on the internet such as YouTube, Kaggle, GitHub etc. to ensure variability in tone, pitch, and environmental conditions.

The extracted audio files were in. MP3 format to ensure consistency and efficient use of storage space. To ensure all audio files are compatible and usable for analysis, the dataset underwent several preprocessing steps. This uniformity avoids compatibility issues during feature extraction and training. Table 1 shows Dog Mood Audio dataset (DMAD) and Table 2 shows Catus Mood Audio dataset (CMAD).

**Table 1:** Dog mood audio dataset (DMAD).

Dog mood audio dataset	
Sad	Angry
1,000	1,000

**Table 2:** Catus mood audio dataset (CMAD).

Angry	Defense	Fighting	Happy	Hunting mind	Paining
1,000	1,000	1,000	1,000	1,000	1,000

### 3.2 Data augmentation and audio preprocessing

Next, data augmentation techniques (36) enhanced the dataset to handle variations in real-world scenarios. This involved introducing slight modifications to existing audio samples, such as adding controlled background noise or adjusting pitch and tempo.

**Figure 2:**
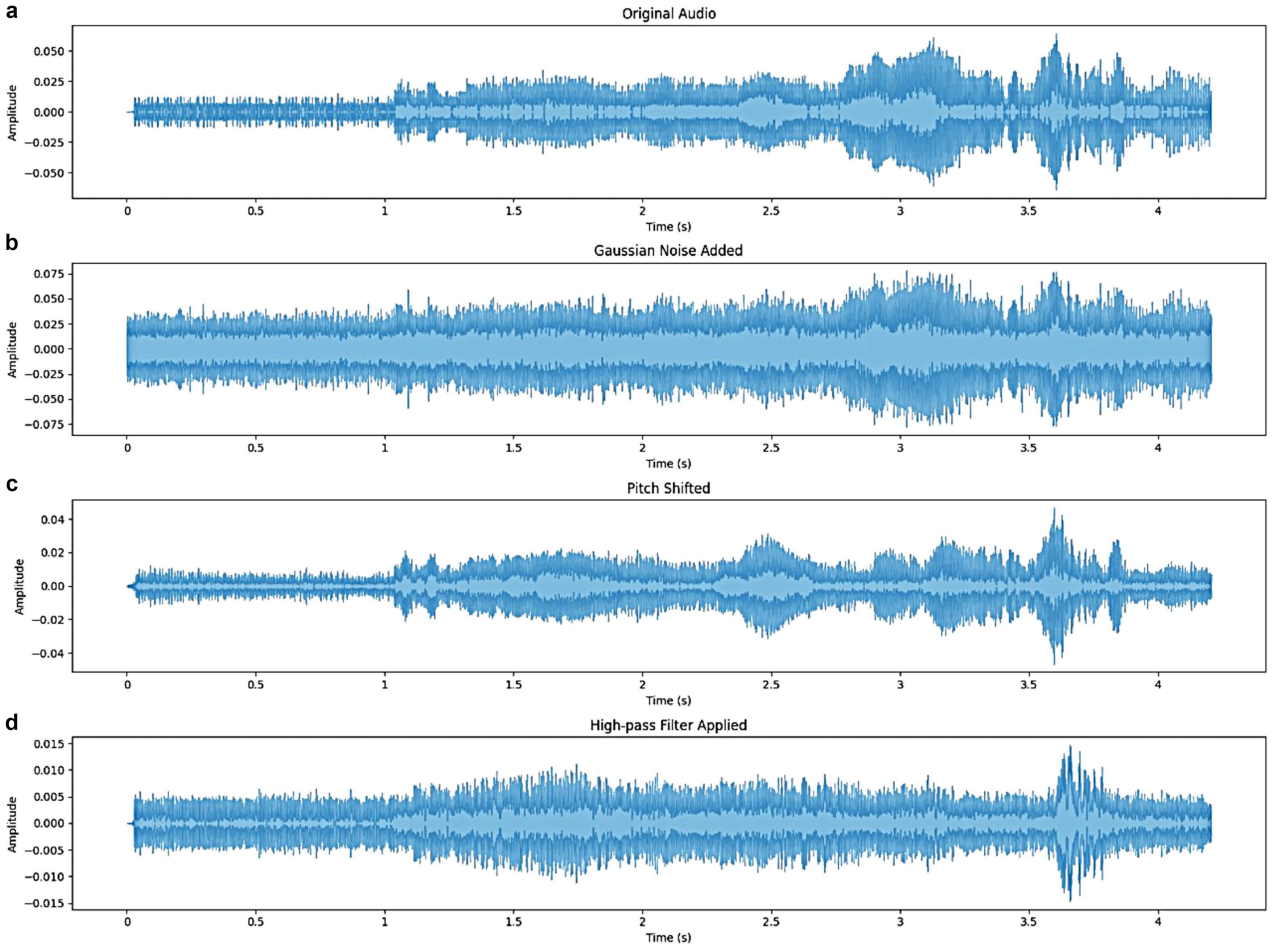
Sample augmented waveform **(a)** original, **(b)** with Gaussian noise added, **(c)** pitch shifted, **(d)** high pass filter applied.

**Figure 3:**
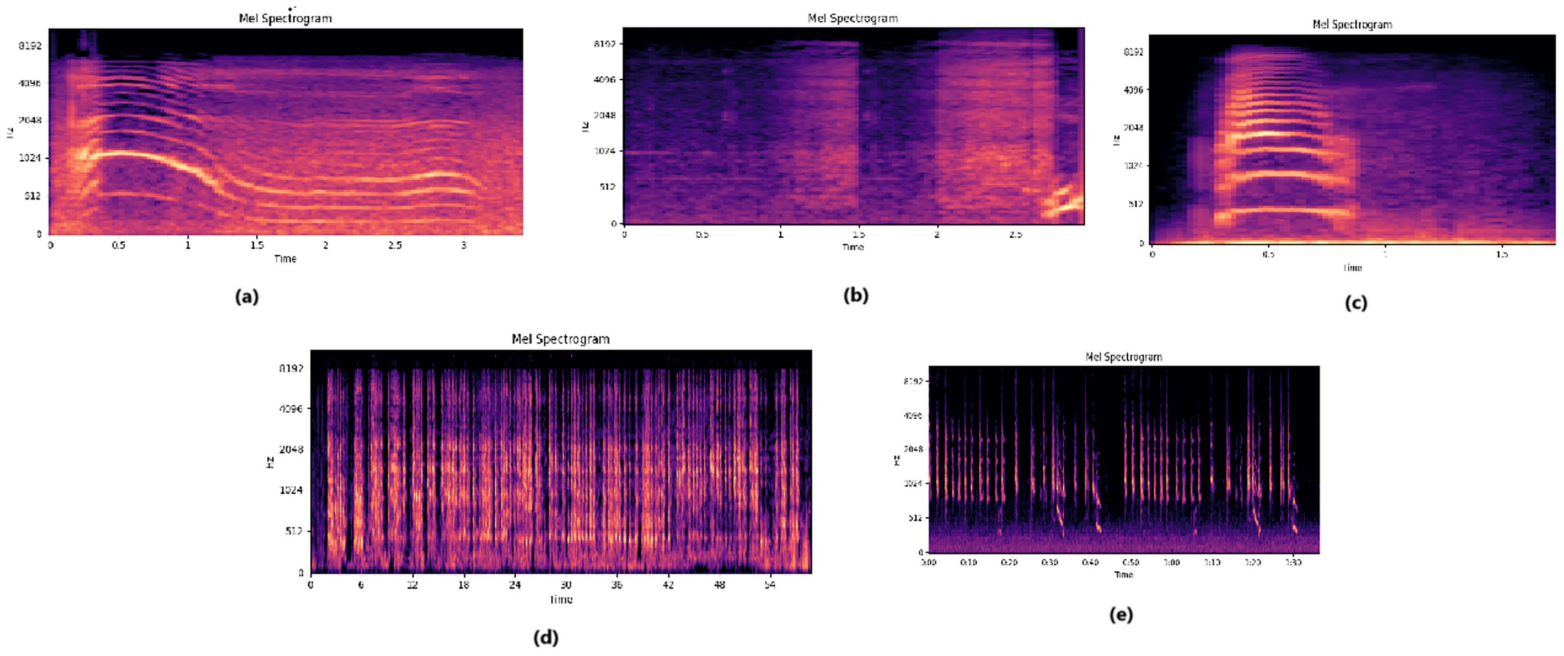
Melspectrogram of CMAD and DMAD audio emotions. Top-row represents cat emotions **(a)** cat-anger **(b)** cat-defense **(c)** cat-happy. Bottom-row represents dog emotions **(d)** dog-angry **(e)** dog-sad.

Data augmentation was applied to the audio data using Audiomentation library. Table 3 shows the non-augmented and augmented CMAD dataset details. Using these libraries, the audio files were loaded, and a sequence of augmentations defined in the augmentations library was applied, and the modified versions were saved as new audio files. Each audio file was augmented by adding Gaussian noise, pitch and by applying high-pass filter. Figure 2 displays the waveforms of original audio and augmented audios. All audio files, including those generated through augmentation, were resampled using the Kaiser fast method to standardize the audio signal frequency. Figure 3 shows the melspectrograms of various pet animal audios. Following this, the resampled audio files were processed using the MFCC (Mel-frequency Cepstral Coefficients) method for feature extraction.

ALGORITHM 1 Proposed multi-QuadEmoNet model.
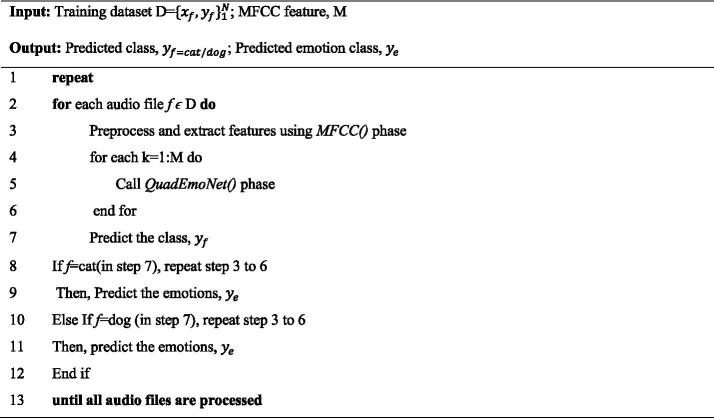

**Table 3:** Before and after augmentation of CMAD details.

Cat emotions	Angry	Defense	Fighting	Happy	Hunting mind	Paining
Before augmentation	50	50	50	50	50	50
After augmentation	1,000	1,000	1,000	1,000	1,000	1,000

In Algorithm 1, for each of the files, f was processed from the training Dataset, D. Each file, f was represented as ${\left\{x_{f},y_{f}\right\}}_{1}^{N}$ . Initially, the dataset was passed through preprocessing and *MFCC()* to extract features.

### 3.3 Feature extraction using MFCC

ALGORITHM 2 Extract MFCC features.
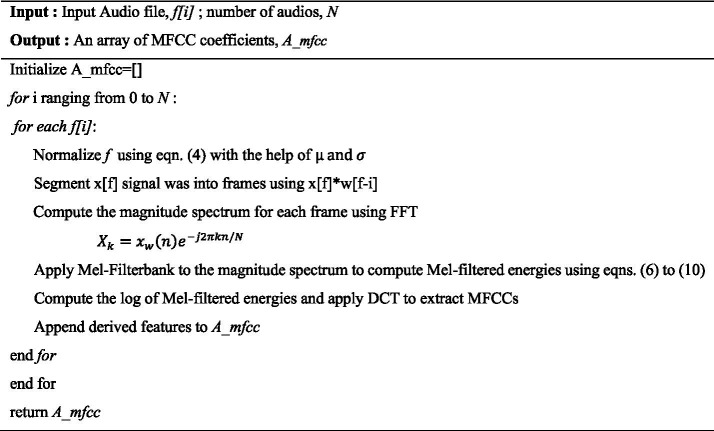

**Figure 4:**
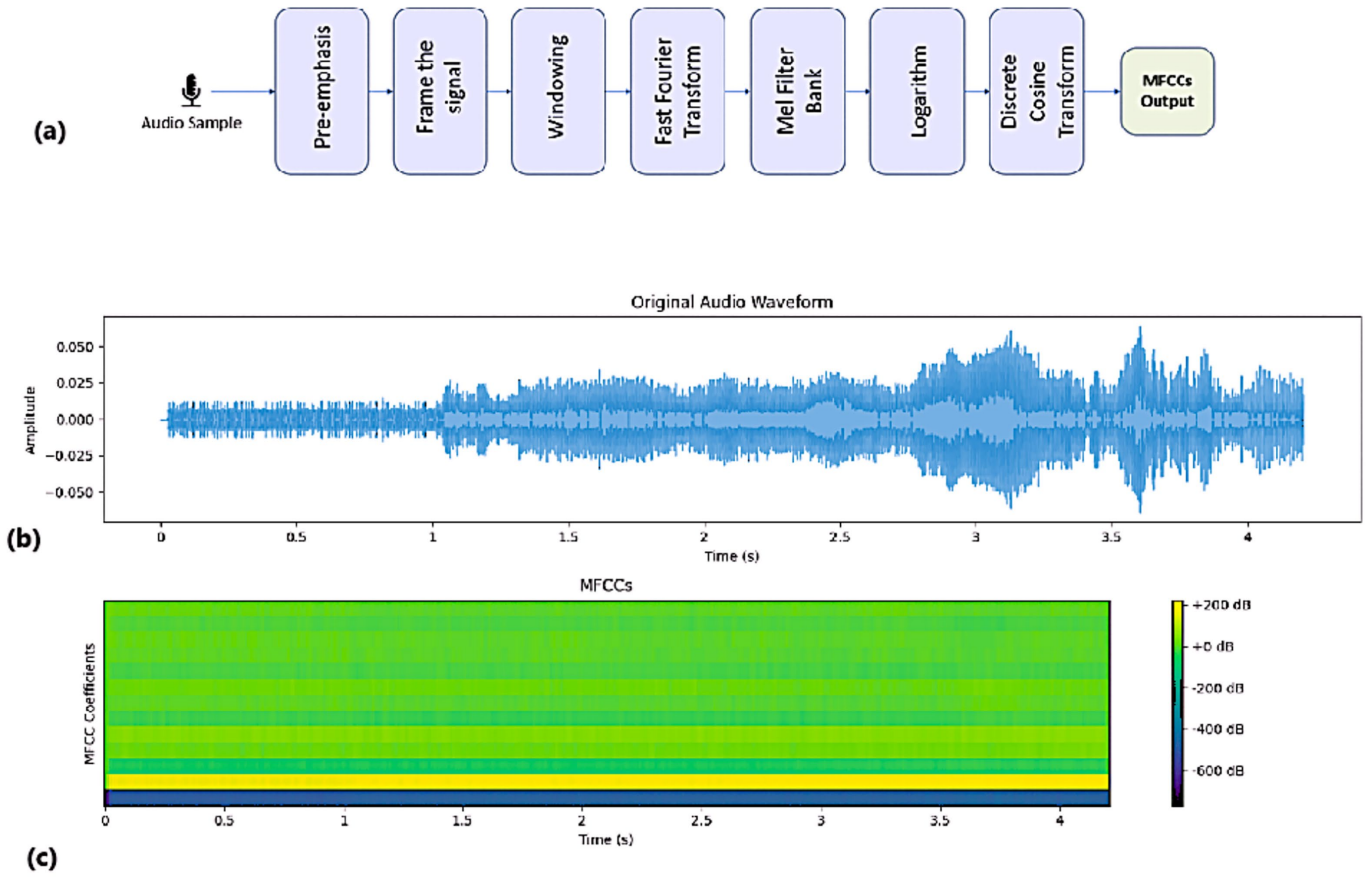
Audio processing using MFCC: **(a)** MFCC architecture, **(b)** original audio, and **(c)** post-MFCC processed audio.

In this paper, Mel-Frequency Cepstral Coefficient (MFCC) serves as a pivotal feature extraction technique for analysing the resampled animal voices. As shown in Figure 4a, the feature extraction using MFCC includes the steps such as pre-emphasis, framing the signal, windowing, Fast Fourier Transform (FFT), Mel-Filter Bank, Logarithm, Discrete Cosine Transform (DCT) begins with signal pre-processing, including normalization and noise reduction, ensuring uniformity and quality. The steps for feature extraction are mentioned in Algorithm 2.

The normalized signal x[f]_normalized_ was computed using the in Equation 1 as follows:


$$x{\left[f\right]}_{\text{normalized}}=\frac{\left(x[f]-\mu \right)}{\sigma }$$ (1)

Where μ represents the mean and σ represents the standard deviation of the signal.

Audio signals are then segmented into frames using a windowing technique. Each frame x_i_[f] was obtained by multiplying the original signal x[f] with a window function w[f-i] as in eqn. 2:


$$x_{i}[f]=x[f]\times w[f-i]$$ (2)

This was followed by the application of Fast Fourier Transform (FFT) for frequency domain conversion.

The Mel Filterbank was employed to transform audio signals from the time domain to the frequency domain, generating filterbank energies that serve as features for further analysis. The filterbank energies H_m_[k] are computed using the triangular filters, where f(m) represents the m^th^ Mel frequency:


$$H_{m}[k]=0\;\text{if}\;k<f(m-1)$$ (3)

$$H_{m}[k]=\frac{k-f(m-1)}{f(m)-f(m-1)}\;\text{if}\;f(m-1)\leq k<f(m)$$ (4)

$$H_{m}[k]=1\;\text{if}\;k=f(m)$$ (5)

$$H_{m}[k]=\frac{f(m+1)-k}{f(m+1)-f(m)}\;\text{if}\;f(m)<k\leq f(m+1)$$ (6)

$$H_{m}[k]=0\;\text{if}\;k>f(m+1)$$ (7)

Here, in Equations (3)–(7), m denotes the index of the Mel filter, and k represents the discrete frequency bin corresponding to the Fourier transform of the signal. Also, f(m) represents center frequency, f(m-1) denotes the lower frequency and f(m + 1) represents the upper frequency.

Log compression and Discrete Cosine Transform (DCT) were sequentially applied to derive the final MFCCs. A subset of coefficients was selected, forming feature vectors for each audio frame. These vectors were concatenated to build feature matrices for individual audio samples. Later, the generated MFCC feature matrices were inserted into emotion recognition models like Long Short architectures and SVMs. This robust MFCC extraction process captures essential vocal characteristics, allowing our models to discern emotional nuances in the audio data. The efficiency of MFCC in representing emotional features makes it a key component in our methodology for accurate emotion recognition in animal vocalizations. Figures 4b,c show the original audio before applying MFCC and the audios post-processing using MFCC. Figure 4c was a spectrogram depicting the audio signal based on the animal sound. The spectrogram highlights the frequencies recorded in the catus (meow/purr) or dog (growl/bark) audios, pertaining to identify their emotions.

### 3.4 Model development

Recurrent Neural Networks (RNNs) (6, 30, 35–37) are a type of Artificial Neural Networks that performs especially well for sequential data processing tasks. They are able to identify temporal relationships in the data as they function by keeping track of a hidden state that changes over time as the network examines each input sequentially. It was highly efficient for tasks such as time series prediction, natural language processing, and analyzing audio signals (30). Traditional feedforward neural networks struggle with capturing the dependencies and the complex temporal relationships present in audio data (31) because they treat each input independently, disregarding the sequential nature of the data. On the other hand, RNNs, with their recurrent connections, can retain information from previous inputs, enabling them to capture patterns and contextual nuances over time.

Long Short-Term Memory (LSTM) networks use gating mechanisms and contain memory cells to process the information inside the network. The input gate was a crucial component and decides if the raw information has to be processed further or not based on the activation function. The input gate uses a sigmoid activation function to output values between 0 and 1. The forget gate controls whether data from the previous time step that was kept in the memory cell was further utilized or discarded. The forget gate consists of one sigmoid activation function, assesses the significance of prior knowledge, and enables the LSTM to selectively recall or forget particular elements based on the most essential task.

Lastly, the information transfer from the memory cell to the LSTM network’s output was controlled by the output gate. The output gate creates the final output of the LSTM by combining data from the current input with the contents of the memory cell. LSTM contains two states, namely, Cell State and Hidden state. Both states of LSTM are described with the help of the following equations.


$$C_{t}=f_{t}(C_{t}-1)+i_{t}(C_{t})$$ (8)

Where, in equation (8) *C_t_* was the current cell state at time step *t*, *f_t_* was the forget gate output at time step *t* (between 0 and 1), it was the input gate output at time step *t* (between 0 and 1) and *C_t_* was the candidate cell state at time step *t*.


$$h_{t}=(O_{t})\times \tanh(C_{t})$$ (9)

Where, in equation (9) *h_t_* was the hidden state (output) at time step *t*, *O_t_* was the output gate output at time step *t* (between 0 and 1), tanh was the hyperbolic tangent activation function and *C_t_* was the current cell state at time step *t*. Each gate (forget, input, output) uses a sigmoid activation function (*σ*) to produce a value between 0 and 1, indicating how much information to allow through. The gates depend on the previous hidden state (h_t_-1), current input (x_t_), and their respective weight matrices and bias vectors.

In equation (10), gate output (*f_t_, i_t_, o_t_*) was given by,


$$g_{t}=\sigma \times (W\times h_{t-1}+U_{i}\times X_{t}+b)$$ (10)

Where, *g_t_* was the generic gate output (*f* for forget*, i* for input, *o* for output), *W* and *U_i_* are the weight matrices for the specific gate, *h_t-1_* was the previous hidden state at time step *t-1*, *x_t_* was the current input at time step *t* and *b* was the bias vector for the specific gate.

Gated Recurrent Units (GRUs), introduced by Cho et al. in 2014, was a variant of recurrent neural networks (RNNs) that employ gating mechanisms to govern the flow of information across time steps. The key idea was to preserve relevant information while removing less important details, thereby enhancing efficiency in sequence modeling. Unlike LSTMs, GRUs simplify the architecture by consolidating components and relying on only two gates: the update gate and the reset gate.

The GRU utilizes two key gates to govern the flow of information across time steps. The update gate (*z_t_*​) sets how much of the previous hidden state should be retained for the next step, while the reset gate (*r_t_*​) identifies the extent to which past information should be removed. By combining these mechanisms, GRUs manage long-term and short-term dependencies more efficiently than traditional RNNs, which rely solely on the hidden state without any gating structure.

The proposed model ‘Multi-QuadEmoNet’ was designed based on the concept of Recurrent Neural Networks, specifically combination of LSTM and GRU to recognize and differentiate between the two different emotional states that dogs and cats express through their vocalizations. Prior to developing the proposed model, several deep learning and machine learning models for created and analyzed. Subsection 3.4.1 discusses the deep learning models. Subsection 3.4.2 discusses the proposed Multi-QuadEmoNet.

#### 3.4.1 Deep learning models

Various models were constructed to identify the best-performing architecture for emotion recognition. The architectures built up for the analysis are illustrated in Figure 4. The different type of models considered for the analysis are single-layered LSTM model, two-layered LSTM model, three layered LSTM model, bi-directional LSTM model, 1D-CNN and proposed QuadEmoNet model, respectively.

##### 3.4.1.1 Single-layered LSTM model

**Figure 5:**
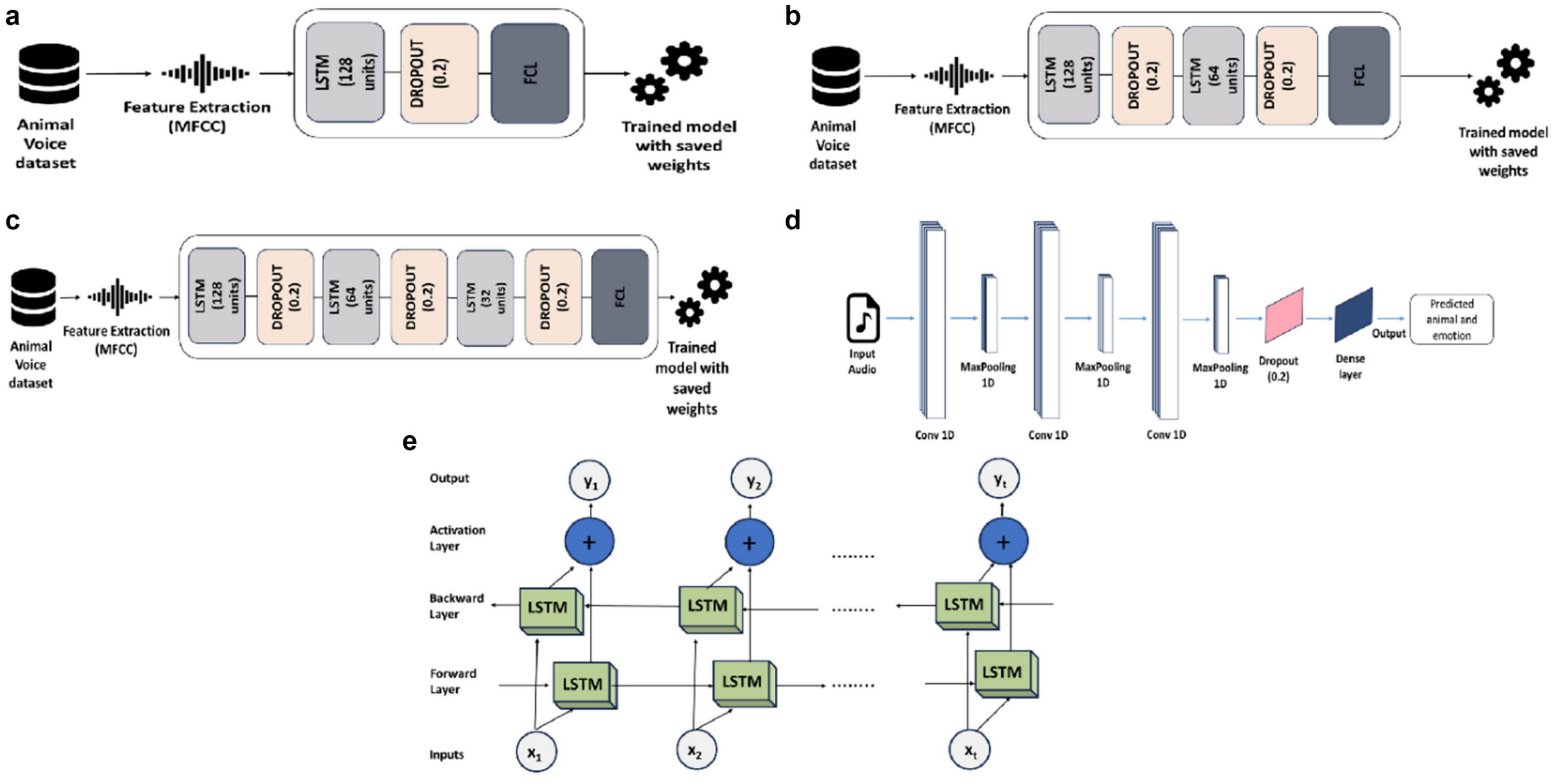
Deep learning models: **(a)** Single-layered LSTM model, **(b)** two-layered LSTM model, **(c)** three-layered LSTM, **(d)** 1D-CNN, **(e)** bi-directional LSTM.

This model employs a single LSTM layer to identify emotional patterns within the audio. Figure 5a shows the single-Layered LSTM layer model. It contains one LSTM layer and one FCL. Single Dropout layer was included between the two layers to tackle overfitting. The benefit of this architecture lies in its relative simplicity, leading to faster training times. However, compared to multi-layered models, it might be less adapted at capturing the nuances of complex emotional dynamics present in animal vocalizations.

##### 3.4.1.2 Two-layered LSTM model

This model employs a two LSTM layers to identify emotional patterns within the audio. It contains two LSTM layer and one FCL. This model was shown in Figure 5b Single Dropout layer was included between the two layers to tackle overfitting. The benefit of this architecture lies in its relative simplicity, leading to faster training times. However, compared to multi-layered models, it might be less adapted at capturing the nuances of complex emotional dynamics present in animal vocalizations.

##### 3.4.1.3 Three-layered LSTM model

This model leverages LSTMs (9), to capture the temporal dependencies inherent in audio sequences. Figure 5c shows the three-layered LSTM. This model was made up of three LSTM layers and one Fully Connected Layer (FCL) making it a three-layered LSTM model.

The first two LSTM layers work together to effectively learn complex emotional patterns from the audio data. Dropout layers are strategically incorporated to mitigate overfitting during the training process. Finally, one fully connected layer was appended at the end for classification. This architecture offers a compelling balance between model complexity and efficiency, making it a strong contender for accurate emotion recognition.

##### 3.4.1.4 Bi-directional LSTM model

Bi-directional LSTM (BLSTM) (30–33) contains a two-way LSTM was utilized for the comparative analysis and was depicted in Figure 5d. Since the audio data was processed in both the forward and backward directions through the BLSTM network, more feature information can be extracted. Two BLSTM layers with Dropout layers and a FCL are involved in this network. The bidirectional architecture holds promise for superior emotion recognition compared to standard LSTMs due to its ability to extract richer contextual features.

##### 3.4.1.5 1D-CNN model

1D-CNN (2, 3) was employed to extract salient features from the audio data. Since the inputs are audio sequences, 1D convolutional layers support the emotion recognition system. The MFCC features were passed to 1D-CNN. Dropout layers are incorporated to mitigate overfitting. While CNNs excel at feature extraction, they might be less sensitive to long-term dependencies compared to LSTMs, which are specifically designed to handle sequential data. Figure 5e shows the 1D CNN model.

##### 3.4.1.6 QuadEmoNet model

**Figure 6:**
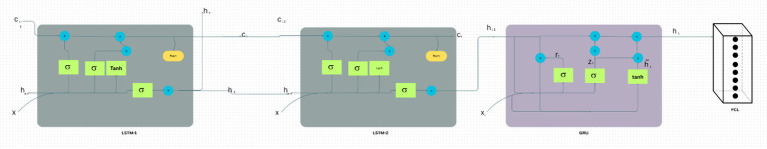
QuadEmoNet architecture.

Figure 6 shows the QuadEmoNet model. Building upon the foundation of the LSTM model, this architecture introduces an additional LSTM layer. So, QuadEmoNet contains two layers of LSTM, one GRU and one FCL. This potentially increases the model’s learning capacity, integrates the feature of both LSTM and GRU enabling it to capture even more intricate emotional patterns within the audio data. Dropout layers remain an essential component to prevent overfitting.

#### 3.4.2 Proposed multi-QuadEmoNet model

A wide analysis was performed using deep learning models. Based on the analysis and comparison of each model, Multi-QuadEmoNet model was designed. The proposed Multi-QuadEmoNet contains three individual QuadEmoNets as shown in Figure 1. Every individual QuadEmoNet was same as in Figure 6. At the primary level, one QuadEmoNet for detecting animal (dog/cat) sounds was utilized. At the secondary level, two QuadEmoNets are utilized to detect dog emotions and cat emotions separately.

The design of QuadEmoNet consists of two Long Short-Term Memory (LSTM) layers, dropout layers to reduce overfitting, and a dense layer for processing at the end. As observed in Figure 5, every individual QuadEmoNet contains a specific similar structure. The first layer of LSTM processes MFCC data, comprising of 64 units. Within the LSTM layer, these units stand for discrete computing components that are individually in charge of identifying particular patterns in the sequential data, like subtleties in audio recordings. A dropout layer was included after the initial LSTM layer to improve generalization by randomly deactivating 30% of neurons during training.

The data processing from the first layer was continued by the second LSTM layer, which has 128 units and improves the representation of sequential patterns. In a similar manner, a dropout layer was added to prevent overfitting before the 128-unit GRU layer further refines the second layer’s processed data. The capacity of the model to capture and understand sequential information, which was essential for identifying intricate patterns in audio recordings. It was enhanced by each unit in these layers. Lastly, the learnt features for the classification task are consolidated by the Fully Connected Layer (FCL), which compiles the data from the GRU layer. QuadEmoNet network’s capacity to comprehend sequential input was improved by its layered design and dropout regularization, which makes it a good choice for tasks like identifying emotions in animal vocalizations. The training process involved for QuadEmoNet was mentioned in Algorithm 3.

ALGORITHM 3 Training single QuadEmoNet model.
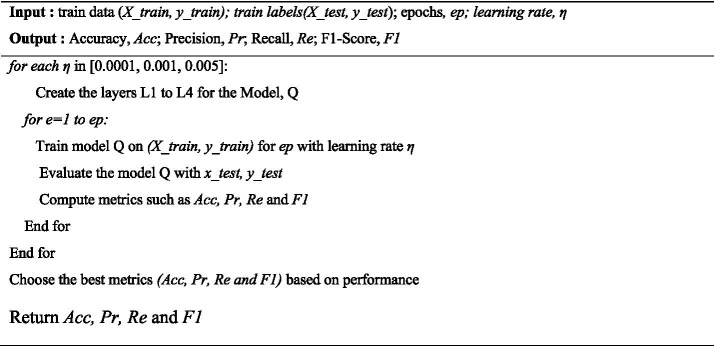

## 4 Results and discussion

The self-collected audio dataset was split in the ratio of 80:20. The proposed Multi-QuadEmoNet and other methods are experimented using python 3.10.4 version. The software packages employed for this research are NumPy, Pandas, sklearn, tensorflow, streamlit and librosa. The python code was implemented on Intel core i5 processor with 8 GB RAM. As mentioned in the previous section, machine learning and deep learning approaches are used for the analysis. Subsection 4.1 contains the evaluation metrics. While subsection 4.2 discusses Machine learning classifiers are discussed, in subsection 4.3, deep learning models are analyzed.

### 4.1 Performance assessment

For evaluating the proposed method and other models, the performance metrics used for the analysis are accuracy, precision, recall, f1-score and confusion matrix. The major elements of these metrics are True Positive (TP), True Negative (TN), False Positive (FP) and False Negative (FN). TP (True Positive) is the Number of samples correctly classified as positive. TN (True Negative) denotes the number of samples correctly classified as negative. FP (False Positive) depict the number of samples incorrectly classified as positive. FN (False Negative) shows the number of samples incorrectly classified as negative.

Accuracy score (Eqn. 11) is defined as the proportion of correctly classified audio recordings. It was otherwise defined as sum of True Positives and False Positives to that of number of Test samples.


$$\begin{array}{c} \text{Accuracy},\mathrm{Acc}=(\text{Number of Correct Predictions})\\ /(\text{Total Number of Test Samples}) \end{array}$$ (11)

Precision (Eqn. 12) measures the proportion of true positives among predicted positives. This reduces the chance of obtaining more FPs.


Precision,Pr=TP/((TP+FP)) (12)

F1-score (Eqn. 13) was the harmonic mean of precision and recall, combining both metrics. For multi-class classification task, F1-score was widely helpful for obtaining conclusion based on the results.


F1−score,F1=2∗((Pr∗Re))/((Pr+Re)) (13)

Recall (Eqn. 14) measures the proportion of true positives identified by ignoring false negatives. This metric explains how good the models are correctly predicting True Positives.


Recall,Re=TP/((TP+FN)) (14)

Confusion matrix: Visualization tool that shows how many recordings from each emotion class were predicted into each category.

### 4.2 Results using machine learning classifiers

CMAD and DMAD are evaluated with four machine learning models, namely, Support Vector Machine (SVM), XGBoost Classifier, LightGBM and CatBoost. The results revealed promising potential for machine learning in deciphering animal emotions as given in Table 4. CatBoost emerged as the frontrunner, achieving an impressive accuracy score of 92% for DMAD and 88% for CMAD.

**Table 4:** Machine learning classifiers.

Model name	SVM		XGB classifier		L:ightGBM		Catboost	
Dataset	DMAD	CMAD	DMAD	CMAD	DMAD	CMAD	DMAD	CMAD
Accuracy score (%)	82.5	56	92	87	94	87	**92**	**88**
Precision	0.84	0.55	0.92	0.87	0.94	0.87	**0.92**	**0.88**
F1 score	0.82	0.55	0.92	0.87	0.94	0.87	**0.92**	**0.88**
Recall	0.82	0.55	0.92	0.87	0.94	0.87	**0.92**	**0.88**

Table shows the highest accuracies in bold.

Furthermore, CatBoost exhibited strong precision (0.92) and recall (0.92) values for DMAD, demonstrating its ability to accurately identify true positives (correctly classifying emotional states) and minimize both false positives (incorrect classifications) and false negatives (missing true emotions). Following closely behind was LightGBM achieving accuracy scores of 94% for DMAD and 87% for CMAD. These models also displayed comparable precision, F1 score, and recall values, suggesting their competence in emotion classification. This suggests that multiple machine learning approaches may be well-suited for this task, with the optimal choice potentially depending on factors like dataset characteristics, computational resources, and interpretability needs.

### 4.3 Results using deep learning

In this section, all possible LSTM models are discussed along with the proposed QuadEmoNet. Deep learning models investigated in this subsection include single layered LSTM layer, 1D-CNN, BLSTM and two layered LSTM.

#### 4.3.1 Results of single-layered LSTM model

The single-layered LSTM model’s performance was evaluated on both the DMAD and CMAD datasets across varying learning rates. Single-layered LSTM model’s performance on the two datasets was shown in Table 5. The results significantly showed that consistent accuracy scores on the DMAD dataset, ranging from 92% with corresponding precision, recall, and F1 scores demonstrating stability across learning rates. Conversely, performance on the CMAD dataset showed similar trends, around 87% accuracy scores. Despite these variations, precision, recall, and F1 scores remained relatively steady. A key finding was the influence of the learning rate on specific metrics. While a learning rate of 0.001 produced similar overall accuracy for both CMAD and DMAD datasets. This suggests that a 0.001 learning rate might have aided the model in achieving a better balance between precision and recall compared to 0.0001 and 0.005.

**Table 5:** Performance metrics of single layered LSTM model.

Model name	Single-layered LSTM					
Dataset	DMAD	CMAD	DMAD	CMAD	DMAD	CMAD
Learning rate	**0.0001**		**0.001**		**0.005**	
Accuracy score	91%	74%	**92%**	**87.00%**	90.00%	85.00%
Precision	0.91	0.74	**0.92**	**0.87**	0.90	0.85
F1 score	0.91	0.74	**0.92**	**0.87**	0.90	0.84
Recall	0.91	0.73	**0.92**	**0.87**	0.90	0.84

Table shows the highest accuracies in bold.

#### 4.3.2 Results of 1D-CNN

The 1D CNN model was assessed on the DMAD and CMAD datasets. Table 6 revealed the notable differences in performance. The model achieved a high accuracy of 93% on the DMAD dataset, showcasing strong precision, recall, and F1 scores. In contrast, performance on the CMAD dataset was about 85.92% of accuracy and value of 0.85 was obtained for precision, recall, and F1 scores. These results suggest that the model effectively captured patterns in the DMAD dataset. Though the number of classes were wider in CMAD dataset, the datasets have attained comparable performance with DMAD.

**Table 6:** Performance metrics of 1D CNN.

Model name	1D-CNN	
Dataset	DMAD	CMAD
Accuracy score	93.00%	85.92%
Precision	0.93	0.85
F1 score	0.93	0.85
Recall	0.93	0.85

Table shows the highest accuracies in bold.

#### 4.3.3 Results of Bi-directional LSTM

Three Bi-directional LSTM (BLSTM) models were evaluated with different learning rates. Table 7 showed the results of BLSTM model with varied learning rates (0.0001, 0.001 and 0.005). All models achieved promising test accuracy ranging from 92 to 94% for DMAD and 82 to 88% for CMAD, demonstrating the capability of BLSTMs to distinguish between animal emotions. However, a crucial finding emerged as a balancing act between overall accuracy and identifying all emotions equally well. The model with the highest accuracy (94%) tended to favor the majority emotion class. While both precision and recall for both classes were high (0.94).

**Table 7:** Performance metrics of bidirectional LSTM.

Model name	Bidirectional LSTM layer					
Dataset	DMAD	CMAD	DMAD	CMAD	DMAD	CMAD
Learning rate	**0.0001**		**0.001**		**0.005**	
Accuracy score	92%	82.00%	93.00%	88.00%	**94%**	**88.00%**
Precision	0.92	0.82	0.93	0.88	**0.94**	**0.88**
F1 score	0.92	0.82	0.93	0.88	**0.94**	**0.88**
Recall	0.92	0.82	0.93	0.88	**0.94**	**0.88**

Table shows the highest accuracies in bold.

The other two BLSTMs (learning rates 0.001 and 0.0001) showcased a trade-off. While their overall accuracy was slightly lower (92 and 93% for DMAD respectively), they exhibited a more balanced distribution of identified emotions in the confusion matrix. These models achieved comparable precision and recall for both emotion classes, indicating good performance across the board.

#### 4.3.4 Results of two-layered LSTM model

The model with two LSTM layers was evaluated on two distinct datasets: DMAD and CMAD, while varying the learning rates. Table 8 displayed the results of two-layered LSTM model. When examining the impact of learning rates on accuracy, it becomes evident that the DMAD dataset exhibits consistent performance across different learning rates, with 93% accuracy for a learning rate of 0.001. Conversely, the CMAD dataset demonstrates sensitivity to the choice of learning rate, with the highest accuracy of 88% achieved at a lower learning rate of 0.001.

**Table 8:** Performance metrics of dual LSTM.

Model name	Two-layered LSTM					
Dataset	DMAD	CMAD	DMAD	CMAD	DMAD	CMAD
Learning rate	**0.0001**		**0.001**		**0.005**	
Accuracy score	87%	78.00%	**93.00%**	**88.00%**	89%	86.00%
Precision	0.88	0.79	**0.93**	**0.89**	0.89	0.86
F1 score	0.87	0.78	**0.93**	**0.88**	0.89	0.86
Recall	0.87	0.78	**0.93**	**0.88**	0.89	0.86

Table shows the highest accuracies in bold.

#### 4.3.5 Three-layered LSTM

Table 9 displays the three-layered LSTM model’s performance metrics at various learning rates for 50 epochs. Using three-layered LSTM, DMAD dataset attained an accuracy of 94% for a learning rate of 0.0001. While for CMAD dataset, an accuracy of 87% was achieved for a learning rate of 0.005.

**Table 9:** Performance of three-layered LSTM.

Model name	3 LSTM layers					
Dataset	DMAD	CMAD	DMAD	CMAD	DMAD	CMAD
Learning rate	**0.0001**		**0.001**		**0.005**	
Accuracy score	**94.00%**	76.00%	93%	85.00%	90%	**87.00%**
Precision	**0.94**	0.76	0.93	0.85	0.89	**0.87**
F1 score	**0.94**	0.76	0.93	0.85	0.89	**0.87**
Recall	**0.94**	0.76	0.93	0.85	0.89	**0.87**

Table shows the highest accuracies in bold.

#### 4.3.6 Proposed QuadEmoNet model

Table 10 showcased the results of QuadEmoNet for 50 epochs. The evaluation of the QuadEmoNet model on the DMAD and CMAD datasets, utilizing different learning rates, unveiled distinct patterns in performance. Notably, higher learning rates fostered enhanced accuracy on the DMAD dataset, peaking at 95% with a rate of 0.005. Conversely, the CMAD dataset displayed sensitivity to learning rates, with optimal accuracy of 90% observed at 0.005. Across both datasets, precision, recall, and F1 scores mirrored the trends in accuracy. Interestingly, models with slightly lower accuracy (around 87%) exhibited a better balance between identifying both emotions. This suggested that a balance between model complexity and learning rate might be crucial for handling datasets with potential class imbalances.

**Table 10:** Performance metrics of proposed QuadEmoNet model.

Model name	QuadEmoNet model					
Dataset	DMAD	CMAD	DMAD	CMAD	DMAD	CMAD
Learning rate	**0.0001**		**0.001**		**0.005**	
Accuracy score	88.00%	88.00%	93	87	**95**	**90**
Precision	0.88	0.88	0.93	0.87	**0.95**	**0.90**
F1 score	0.88	0.88	0.93	0.87	**0.95**	**0.90**
Recall	0.88	0.88	0.93	0.87	**0.94**	**0.90**

Table shows the highest accuracies in bold.

Table 11 shows the performance of QuadEmoNet when datasets were used to classify if the audio was from cat or dog. An accuracy of 95% was attained for classifying cat audios and dog audios with a learning rate of 0.005 using Adam optimizer (50 epochs). At the secondary level, emotions were classified. As a result, the decision was made to handle the CMAD and DMAD datasets separately using QuadEmoNet, allowing the model to better optimize for the unique characteristics of each dataset.

**Table 11:** Performance metrics of level-1 QuadEmoNet using merged datasets for classifying dog or cat pet animal.

Metrics	Merged dataset-level 1 of multi-stage QuadEmoNet
Accuracy	95%
Precision	0.95
Recall	0.95
F1-score	0.95

QuadEmoNet model was evaluated by increasing the number of epochs with the results were presented in Table 12. When the number of epochs was increased, it was observed that the results for DMAD produced an accuracy of 95% at 100 epochs. While for CMAD, an accuracy of 90% was achieved at 50 epochs. Until 100 epochs, accuracy improvement was present. On further increasing after 100 epochs, accuracy was decreased, indicating the optimal number of epochs for CMAD was 50 epochs and while for DMAD, it was 100 epochs.

**Table 12:** Results on increasing number of epochs.

Dataset	Number of epochs	Accuracy	Precision	Recall	F1 score
CMAD	**50**	**90**	**0.90**	**0.90**	**0.90**
	75	89	0.89	0.89	0.89
	**100**	89	0.89	0.89	0.89
DMAD	50	93	0.93	0.93	0.92
	**75**	**95**	**0.95**	**0.95**	**0.95**
	100	94	0.94	0.94	0.93

Table shows the highest accuracies in bold.

### 4.4 Result summary

**Figure 7:**
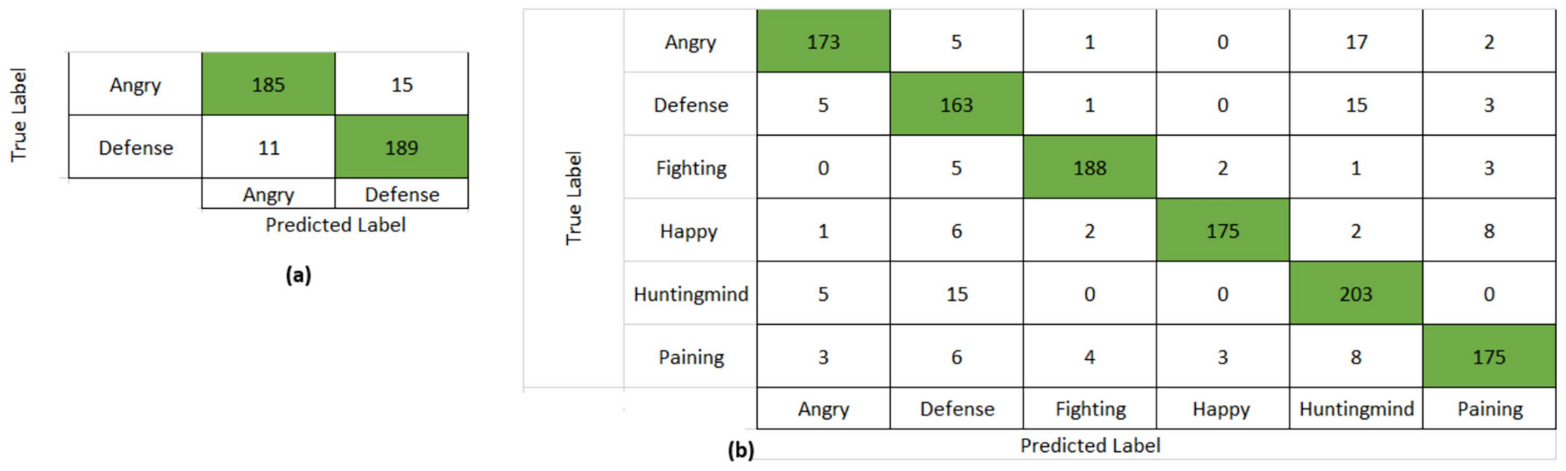
Confusion matrices of **(a)** DMAD dataset **(b)** CMAD dataset.

**Figure 8:**
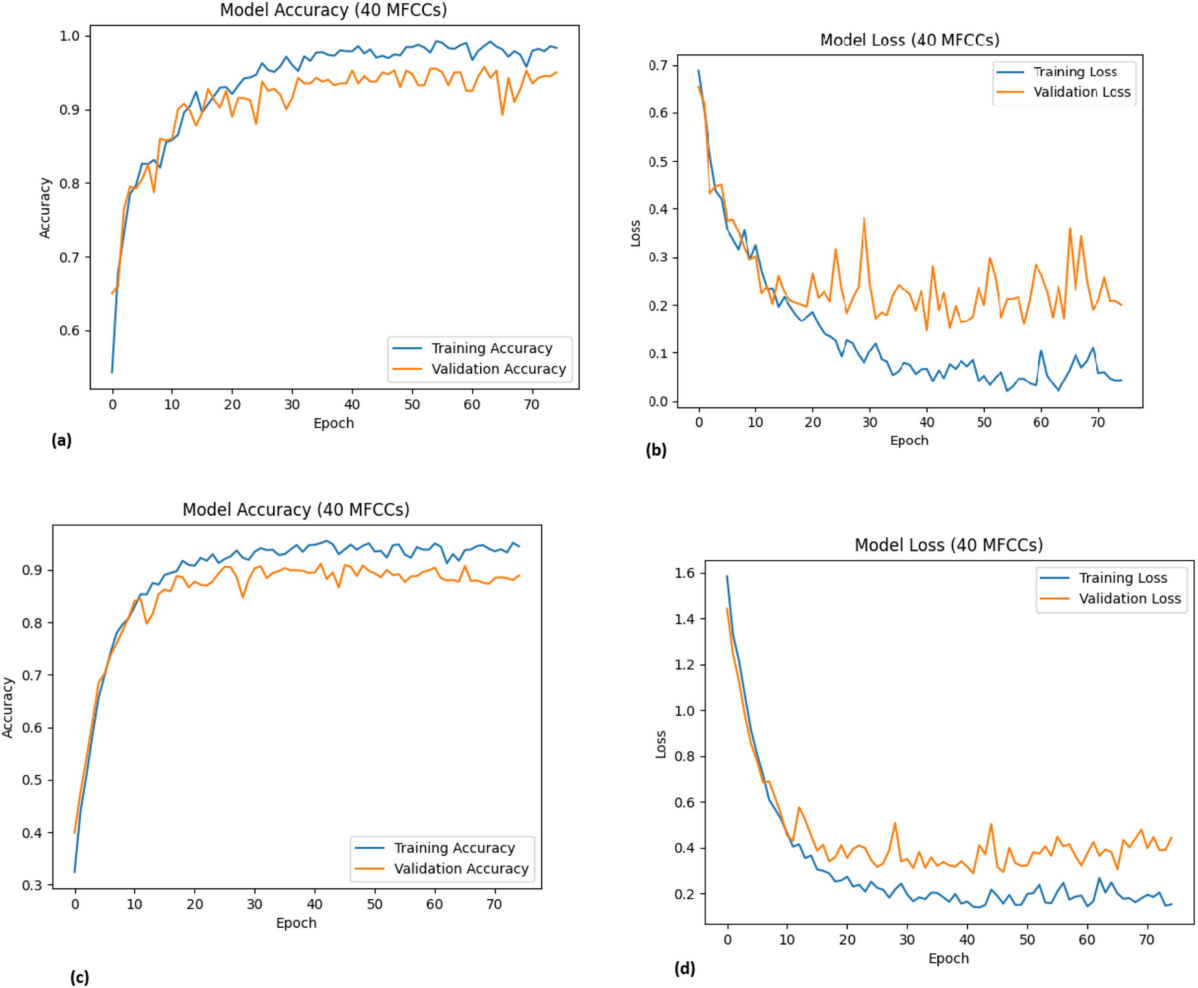
Train and validation loss graph for CMAD **(a,b)** and DMAD **(c,d)** dataset.

Figure 7a shows the confusion matrix of the best-performing model for DMAD, QuadEmoNet model with learning rate 0.005 at 50 epochs and Figure 7b shows the confusion matrix of the best performing model for CMAD, QuadEmoNet model with learning rate 0.005 at 75 epochs. The mispredictions among angry, defense, and hunting mind vocalizations in cats were attributable to their shared arousal-driven origin, which produces vocalizations of similar mid-to-high frequency ranges, comparable intensity levels, and short, abrupt temporal structures. This acoustic convergence is a recognized ethological constraint rather than solely a modeling limitation, highlighting the importance of incorporating contextual cues alongside acoustic features for improved classification. Figures 8a,b illustrated the loss and accuracy curves obtained for CMAD dataset using QuadEmoNet. Figures 8c,d illustrated the loss and accuracy curves obtained for the DMAD dataset using QuadEmoNet.

Based on the results obtained from various models for emotion recognition from audio, it can be inferred that the QuadEmoNet model outperforms the other models in terms of multiple evaluation metrics. This model achieved the highest accuracy score of 95% for DMAD and 90% for CMAD datasets. These higher accuracies indicate the proposed method’s ability to correctly classify animal auditory emotions. With a precision score of 0.95 and 0.90 for DMAD and CMAD datasets. Based on the precision score, it was understood that the proposed method demonstrates a high level accuracy and was helpful in correctly identifying positive instances, minimizing false positives.

The F1-score values of 0.95 and 0.90 suggested a balanced performance between precision and recall. This value revealed that the model was effective in handling both false positives and false negatives. The recall scores of 0.95 and 0.90 indicates the model’s capability to capture a high proportion of actual positive instances, further supporting its effectiveness in recognizing emotions in audio. The confusion matrix for this model shows a balanced distribution of true positives, true negatives, false positives, and false negatives, reflecting a robust performance across different emotion classes.

The McNemar’s test (Table 12) was applied to compare the classification performance of the LSTM model with the proposed QuadEmoNet model on both CMAD and DMAD datasets. On CMAD, the number of cases where QuadEmoNet was correct but LSTM was wrong (94) was greater than the reverse situation where LSTM was correct but QuadEmoNet was wrong (66). The test statistic (66) with a *p*-value of 0.0325 (< 0.05) indicates that this improvement was statistically significant. On DMAD, a similar trend was observed: QuadEmoNet correct, LSTM wrong = 29, compared to LSTM correct, LSTM-GRU wrong = 13. The test statistic (13) with a p-value of 0.0195 (< 0.05) again confirms a significant difference in favor of QuadEmoNet. To conclude, the proposed QuadEmoNet performs well for both CMAD and DMAD datasets (see Table 13).

**Table 13:** McNemar test for CMAD and DMAD datasets.

Contingency table	CMAD	DMAD
Both correct	975	347
Three-layered LSTM correct, proposed QuadEmoNet (LSTM-GRU) incorrect	66	13
Three-layered LSTM incorrect, proposed QuadEmoNet (LSTM-GRU) correct	94	29
Both incorrect	65	11
Statistic	66	13
*p*-value	0.0325	0.0195

The results clearly showed that QuadEmoNet outperformed all baseline models across both DMAD and CMAD datasets. It achieves the highest accuracy (95% on DMAD and 90% on CMAD), precision, recall, and F1-score while maintaining a balanced trade-off between computational efficiency and inference time. Ablation study performed was portrayed in Table 14. Although its inference time (82.47 ms for DMAD and 92.73 ms for CMAD) was slightly higher than simpler LSTM variants, it remains significantly lower than 1D-CNN and comparable to Bi-directional LSTM, with moderate FLOPs (8.58 M). This demonstrates that QuadEmoNet not only delivers superior recognition performance but also ensures computational feasibility, making it the most effective and practical choice among the tested models. The dataset augmentation pipeline (Section 3.2) applies Gaussian noise addition, pitch shifting, and high-pass filtering to training samples. Since the QuadEmoNet model was trained on these augmented variants, the reported accuracy Figures (95% for DMAD, 90% for CMAD) inherently reflect performance under noisy, real-world-like conditions. An inference time of ~82–93 ms per 60 s clip confirmed that the system operated well within real-time constraints for practical deployment in pet monitoring and veterinary applications.

**Table 14:** Ablation study of the proposed QuadEmoNet.

Metrics	DMAD						CMAD					
Model	Accuracy	Precision	Recall	F1-score	Inference time (ms)	FLOPs (M)	Accuracy	Precision	Recall	F1-score	Inference time (ms)	FLOPs
Single layered LSTM	92	0.92	0.92	0.92	31.7636	2.66	87	0.87	0.87	0.87	105.2945	2.66
Dual layered LSTM	93	0.93	0.93	0.93	48.0947	4.64	88	0.88	0.88	0.88	37.3771	4.64
Three layered LSTM	94	0.94	0.94	0.94	52.1948	5.14	87	0.87	0.87	0.87	31.6868	5.14
1D-CNN	93	0.93	0.93	0.93	283.1566	0.25	85.92	0.85	0.85	0.85	362.4218	0.25
Bi-directional LSTM	94	0.94	0.94	0.94	56.5152	11.90	88	0.88	0.88	0.88	60.1358	11.90
Proposed QuadEmoNet	**95**	**0.95**	**0.95**	**0.94**	**82.4735**	**8.58**	**90**	**0.90**	**0.90**	**0.90**	**92.7298**	**8.58**

Table shows the highest accuracies in bold.

#### 4.4.1 Comparison with existing methods

YAMNet (31), CNN10, and CNN14 were used for comparative analysis with the designed QuadEmoNet as showed in Table 15. The CNN-10 model achieved an accuracy of 72.6%, a precision of 0.72, a recall of 0.72, and an F1-score of 0.72 on the CMAD dataset. The CNN14 model achieved an accuracy of 74% lower than that of the proposed QuadEmoNet when evaluated on the CMAD dataset. YAMNet achieved an accuracy of 75%.

In the DMAD dataset, CNN10, CNN14, and YAMNet achieved accuracies of 90, 92, and 93.5%, respectively. In comparison to the current methodologies, the suggested QuadEmoNet demonstrated superior performance on the dog dataset, with an accuracy improvement of at least 1.5% (see Table 15).

**Table 15:** Summary of results.

Method	CMAD				DMAD			
	Accuracy	Precision	Recall	F1-Score	Accuracy	Precision	Recall	F1-score
CNN10	72.5	0.72	0.72	0.72	90	0.90	0.89	0.89
CNN14	74	0.74	0.74	0.74	92	0.92	0.92	0.92
YAMNET	75	0.75	0.75	0.75	93.5	0.94	0.93	0.93
**QuadEmoNet**	**90**	**0.90**	**0.90**	**0.90**	**95**	**0.95**	**0.95**	**0.95**

Table shows the highest accuracies in bold.

#### 4.4.2 Real-time implementation

**Figure 9:**
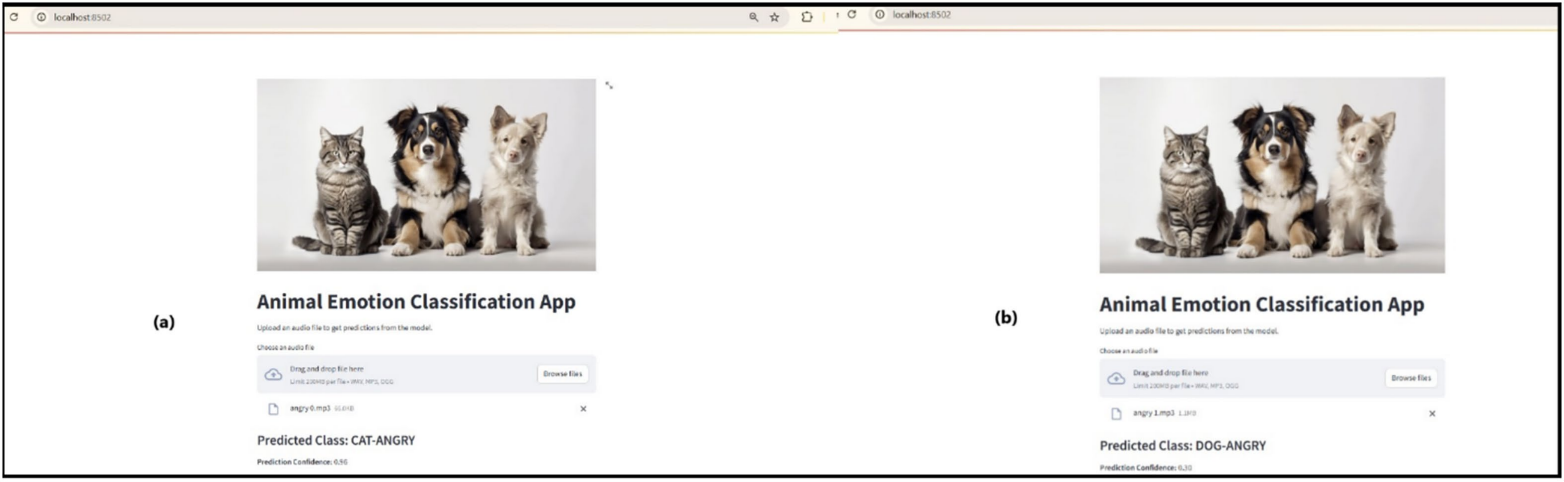
Website application for animal emotion recognition using Multi-QuadEmoNet based on animal audio. Authors’ own work (QuadEmoNet application).

Since, proposed QuadEmoNet outperformed other models, the best trained weights were further used for the real-time implementation using a web application. As part of our research work, an user interface was developed for predicting the emotions of dog or cat based on their audios using proposed Multi-QuadEmoNet model. The web user interface was developed using steamlit application. Figure 9 showcased the website design for pet emotion recognition using QuadEmoNet. In Figures 9a,b shows the audio sample being uploaded on clicking the choose file button. After the audio file was being loaded, the sample audio was tested on the saved model and the resulting animal emotion was displayed as the output.

#### 4.4.3 Limitations and future work

The emotional responses of companion animals (dogs and cats) demonstrate several bottlenecks with respect to dataset limits and the model’s generalizability. The dataset gathered from diverse online audio sources, characterized by different noise levels, may result in errors during feature learning due to differences in audio quality and recording conditions. Another significant limiting factor was the real-world deployment scenario and usability of the bioacoustic tool.

Future work will explore the integration of Multi-QuadEmoNet into wearable devices and mobile applications for continuous, real-time monitoring of animals in clinical settings. Such implementations could enable non-invasive tracking of stress, pain, or discomfort during hospitalization, supporting veterinarians with timely insights and improving animal welfare. Further efforts will focus on optimizing the model for edge deployment, reducing computational overhead, and validating performance in real-world veterinary environments.

## 5 Conclusion

The proposed Multi-QuadEmoNet system aimed to bridge the communication gap between humans and animals by decoding the animal emotions from their vocalizations. This research focused on two primary animal companions – cats and dogs. A dataset containing 6,000 cat vocalizations and 2000 dog vocalizations curated for the datasets CMAD and DMAD. All animal audios were preprocessed. The preprocessed data were fed into Mel-Frequency Cepstral Coefficients (MFCCs) for feature extraction. These features were then employed to train various machine learning and deep learning models for emotion recognition. Among the evaluated models, the QuadEmoNet model emerged as the most successful. For the DMAD, this model achieved a remarkable accuracy score of 95%, along with precision of 0.95, recall of 0.95, and F1-score of 0.95, respectively. For CMAD, this model achieved an accuracy score of 90% along with precision of 0.90, recall of 0.90, and F1-score of 0.90, respectively. These metrics collectively indicate the model’s efficacy in accurately classifying emotions within the audio data using Multi-QuadEmoNet method. This technique has the potential to support various fields, including pet care, veterinary medicine etc. As a potential futurework, QuadEmoNet can be embedded into low-cost edge devices such as Raspberry Pi, integrated with microphones or recorders in pet clinics. This setup would enable real-time inference of animal emotions in practical environments. For pet owners, this system offers the enticing prospect of gaining a deeper understanding of their furry companions’ emotional well-being. By recognizing emotions like happiness, fear, or anxiety, pet owners can tailor their interactions to foster a stronger bond and provide more targeted care.

## Data Availability

The original contributions presented in the study are included in the article/supplementary material, further inquiries can be directed to the corresponding author.
